# Comparison of the characteristics of the population eligible for lung cancer screening under 2013 and population newly eligible under 2021 US Preventive Services Task Force recommendations

**DOI:** 10.1007/s10552-024-01880-6

**Published:** 2024-05-08

**Authors:** Nicholas Yell, Jan M. Eberth, Anthony J. Alberg, Peiyin Hung, Mario Schootman, Alexander C. McLain, Reginald F. Munden

**Affiliations:** 1 Department of Health Services Policy and Management, University of South Carolina Arnold School of Public Health, Columbia, SC USA; 2 Department of Health Management and Policy, Drexel University Dornsife School of Public Health, Philadelphia, PA USA; 3 Department of Epidemiology and Biostatistics, University of South Carolina Arnold School of Public Health, Columbia, SC USA; 4 Department of Internal Medicine, University of Arkansas for Medical Sciences, Little Rock, AR USA; 5 Department of Radiology and Radiological Sciences, Medical University of South Carolina, Charleston, SC USA

**Keywords:** Lung cancer, Disparities, LDCT screening, USPSTF recommendations, Eligibility

## Abstract

**Purpose:**

In 2021, the United States Preventive Services Task Force (USPSTF) revised their 2013 recommendations for lung cancer screening eligibility by lowering the pack-year history from 30+ to 20+ pack-years and the recommended age from 55 to 50 years. Simulation studies suggest that Black persons and females will benefit most from these changes, but it is unclear how the revised USPSTF recommendations will impact geographic, health-related, and other sociodemographic characteristics of those eligible.

**Methods:**

This cross-sectional study employed data from the 2017–2020 Behavioral Risk Factor Surveillance System surveys from 23 states to compare age, gender, race, marital, sexual orientation, education, employment, comorbidity, vaccination, region, and rurality characteristics of the eligible population according to the original 2013 USPSTF recommendations with the revised 2021 USPSTF recommendations using chi-squared tests. This study compared those originally eligible to those newly eligible using the BRFSS raking-dervived weighting variable.

**Results:**

There were 30,190 study participants. The results of this study found that eligibility increased by 62.4% due to the revised recommendations. We found that the recommendation changes increased the proportion of eligible females (50.1% vs 44.1%), Black persons (9.2% vs 6.6%), Hispanic persons (4.4% vs 2.7%), persons aged 55–64 (55.8% vs 52.6%), urban-dwellers(88.3% vs 85.9%), unmarried (3.4% vs 2.5%) and never married (10.4% vs 6.6%) persons, as well as non-retirees (76.5% vs 56.1%) Respondents without comorbidities and COPD also increased.

**Conclusion:**

It is estimated that the revision of the lung cancer screening recommendations decreased eligibility disparities in sex, race, ethnicity, marital status, respiratory comorbidities, and vaccination status. Research will be necessary to estimate whether uptake patterns subsequently follow the expanded eligibility patterns.

**Supplementary Information:**

The online version contains supplementary material available at https://doi.org/10.1007/s10552-024-01880-6.

## Introduction

Lung cancer is the leading cause of cancer death in the United States, causing an estimated 30% of all cancer deaths annually [1]. In the US, based on SEER data the majority of lung cancers are diagnosed with distant stage disease after the cancer has metastasized, when the five-year survival rate is 8.2%, compared with those who are diagnosed with localized disease when the five-year survival rate is 62.8% [2]. In 2011, the results from the National Lung Screening Trial (NLST) demonstrated 20% fewer lung cancer deaths in those randomized to LDCT screening compared with those screened with chest radiography. Subsequent LDCT screening trials such as the Nederlands-Leuvens Longkanker Screenings Onderzoek (NELSON) trial demonstrated similar results [3, 4].

In 2013, based on results of the NLST and other studies, the United States Preventive Services Task Force (USPSTF) recommended annual LDCT screening for individuals who were between 55 and 80 years of age, had a 30+ pack-year smoking history, and currently smoked or had quit within 15 years. In 2021, the USPSTF revised its recommendation to reduce disparities in eligibility by sex and race based on evidence that showed that Black persons and women tend to smoke fewer cigarettes than White men but remain at increased risk for lung cancer [5]. To accomplish this, the age of screening initiation was lowered from age 55 to 50 years and the minimum pack-year smoking history was lowered from at least 30 to at least 20 pack-years [6]. The recommendations were not changed for quitting within 15 years, even though one study found that 40.8% of persons who previously smoked and quit for more than 15 years developed lung cancer [7]. Lung cancer risk continues to decrease with time since quitting, but compared with those who never smoked, the risk of lung cancer remains elevated in persons who formerly smoked for decades [8]. Recent American Cancer Society lung cancer screening guidelines have removed the years since quit criteria accordingly [9].

Simulation studies have shown that Black persons and women would have the greatest increase in LDCT screening eligibility due to the revised recommendations [10], and these results have been replicated with real-world data [11–15]. Another study found increased eligibility among those without a chronic obstructive pulmonary disease (COPD) diagnosis and among those of lower social-economic status measured using the Yost index, which is a composite census tract-level SES measure based on the combination of two heavily weighted variables (household income, poverty) and five less heavily weighted variables (rent, home value, employment, education and working class [16]. Additional studies are needed to replicate these prior findings and to assess whether the revised recommendations increased eligibility among other groups at increased risk for developing or dying from lung cancer, such as other sociodemographic characteristics (e.g., marital status, sexual minority status), health status (e.g., comorbidities), and geographic variables (e.g., urban/rural residence) [17–24]. Therefore, our study characterizes the differences in the distribution of these sociodemographic, health-related, and geographic variables among the screening eligible population according to the original 2013 USPSTF recommendations with those newly eligible based on the new criteria under the revised 2021 USPSTF recommendations (i.e., eligible under the 2021 criteria but *NOT* the 2013 criteria). Understanding these differences will help to better understand the population impact of the change in recommendations, identify population subgroups for targeted outreach to increase LDCT screening uptake because they became newly eligible, and determine geographic areas or populations likely to benefit from additional resources to effectively administer LDCT screening programs.

## Methods

### Data source and study participants

The data for this cross-sectional study comes from the Behavioral Risk Factor Surveillance System (BRFSS). The BRFSS is a health-related, nationally representative telephone survey (cell and landline) conducted by all 50 states, the District of Columbia, Guam, and Puerto Rico. The BRFSS has four questionnaire versions: Common, Version 1, Version 2, and Version 3. The BRFSS has a response rate that ranges from 31 to 64% depending on the state. For each year, all versions that included the optional lung cancer module (i.e., states opt in or out) were used. The lung cancer module was included in all common versions as well as BRFSS 2017 version 2, BRFSS 2018 versions 1 and 2, and BRFSS 2019 versions 1 and 2. This study used survey data from 23 states that included the lung cancer optional module in one or more years of BRFSS 2017–2020 (AZ, DE, FL, GA, ID, KS, KY, MD, ME, MN, MO, MT, NC, ND, NE, NJ, NV, OK, PA, RI, SC, SD, and TX). To compare the changes in the population eligible for LDCT screening, this study only includes those who were eligible under the 2013 and/or 2021 USPSTF recommendations (e.g., ineligible participants are not included). Furthermore, participants who reported they were diagnosed with lung cancer were excluded since lung cancer survivors tend to be on different surveillance protocols.

### Measurements

**Fig. 1:**
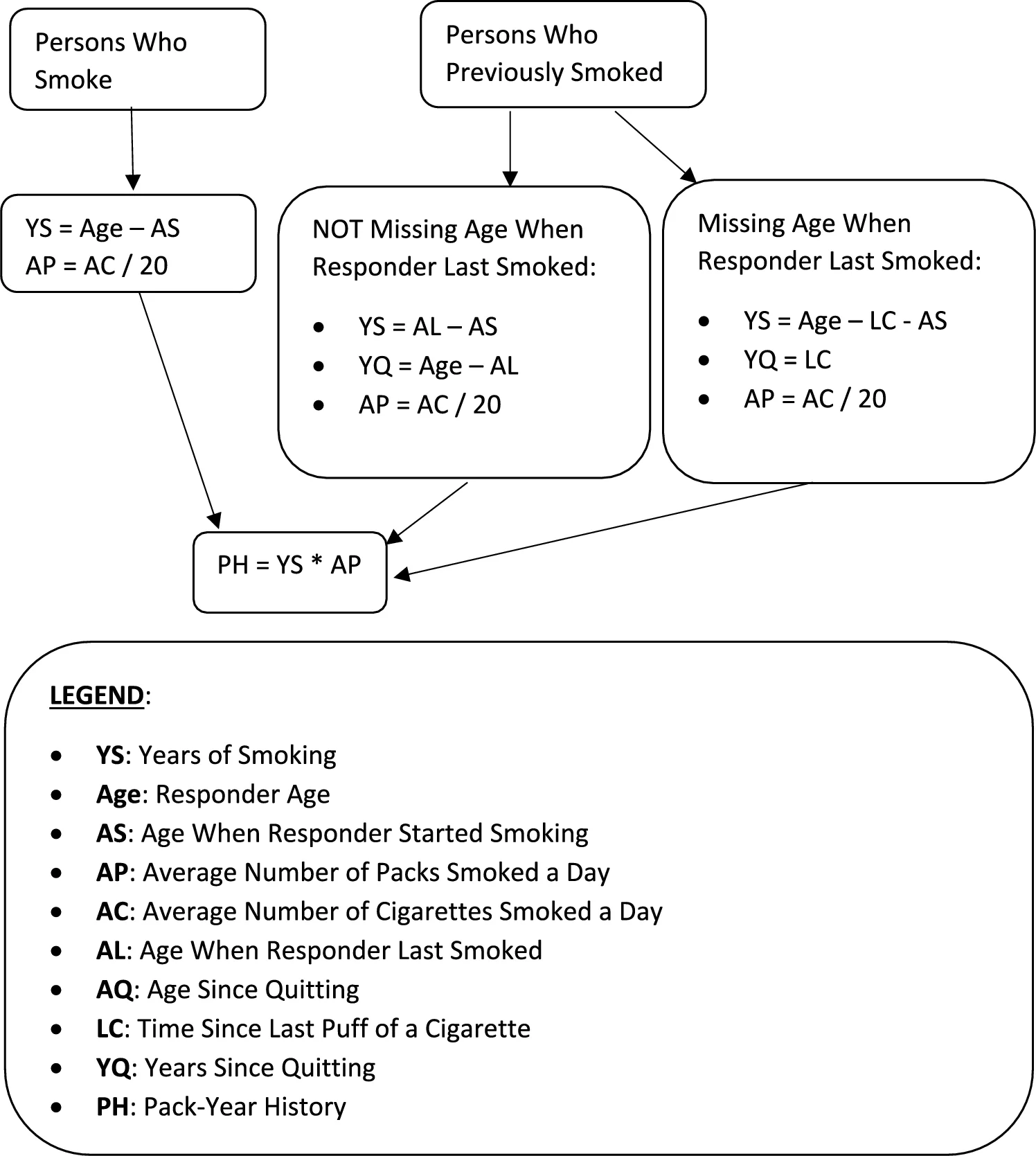
Calculating pack-year history and years since quitting smoking

The study variables used for this study were sociodemographic characteristics, variables related to health status, and geographic variables; these are described in detail below. The primary variable of interest was whether a participant was classified as meeting the 2013 USPSTF recommendations or only the 2021 USPSTF recommendations, which were classified separately. Participants between the ages of 55 and 80 years old, with a 30+ pack-year smoking history, and who currently smoked or quit within the past 15 years were classified as eligible for screening under the 2013 recommendations. Participants meeting the same criteria, except with an age between 50 and 54 and/or a 20–29 pack-year smoking history were classified as eligible under the revised 2021 recommendations only. This study does NOT compare participants eligible under the 2013 USPSTF recommendations against all participants eligible under the 2021 USPSTF recommendations, since the new recommendations were revised to increase eligibility and therefore all participants eligible under the 2013 USPSTF recommendations are also eligible under the new 2021 USPSTF recommendations. The variables used for pack-year smoking history and years since quitting were calculated using the process illustrated in Fig. 1.

### Study variables

Study variables were selected based on potential associations with smoking intensity, frequency, or cessation since they were the key changes in the USPSTF revision [10–28], including sociodemographic, health-related, and geographic variables. If participants were missing data for a variable, they were removed from the calculations relating to that variable.

*Sociodemographic variables* included self-reported age group, sex, race and ethnicity, sexual orientation, marital status, educational status, and retirement status. The *health-related variables* included vaccination status and the presence of respiratory and cardiovascular comorbidities. Respiratory comorbidities included a diagnosis of asthma and/or COPD/emphysema/chronic bronchitis. If a participant reported ever having angina, a heart attack, heart disease, or a stroke, they were considered to have a cardiovascular comorbidity. These variables were included because individuals with these preexisting conditions and cardiovascular comorbidities are more likely to develop lung cancer [29–31]. They were also included because they increase the likelihood of smoking cessation [23, 32]. Vaccination status was classified based on whether the participant had received the pneumonia vaccination ever, the flu vaccination in the past year, both, or neither. Vaccination status was included because it indicates a willingness to engage in prevention activities and provides an opportunity for engagement with a healthcare provider for which other services can also be provided (e.g., cancer screening referrals and smoking cessation). The *geographic variables* included the state of residence at the time of the survey, urban/rural status, and U.S. census region. Urban/rural status identified whether the participant resided in an urban or a rural area based on the 2013 National Center for Health Statistics Urban–Rural Classification Scheme. Region was classified as residing in the Midwest, Northeast, South, or West.

#### Study population definition

**Fig. 2:**
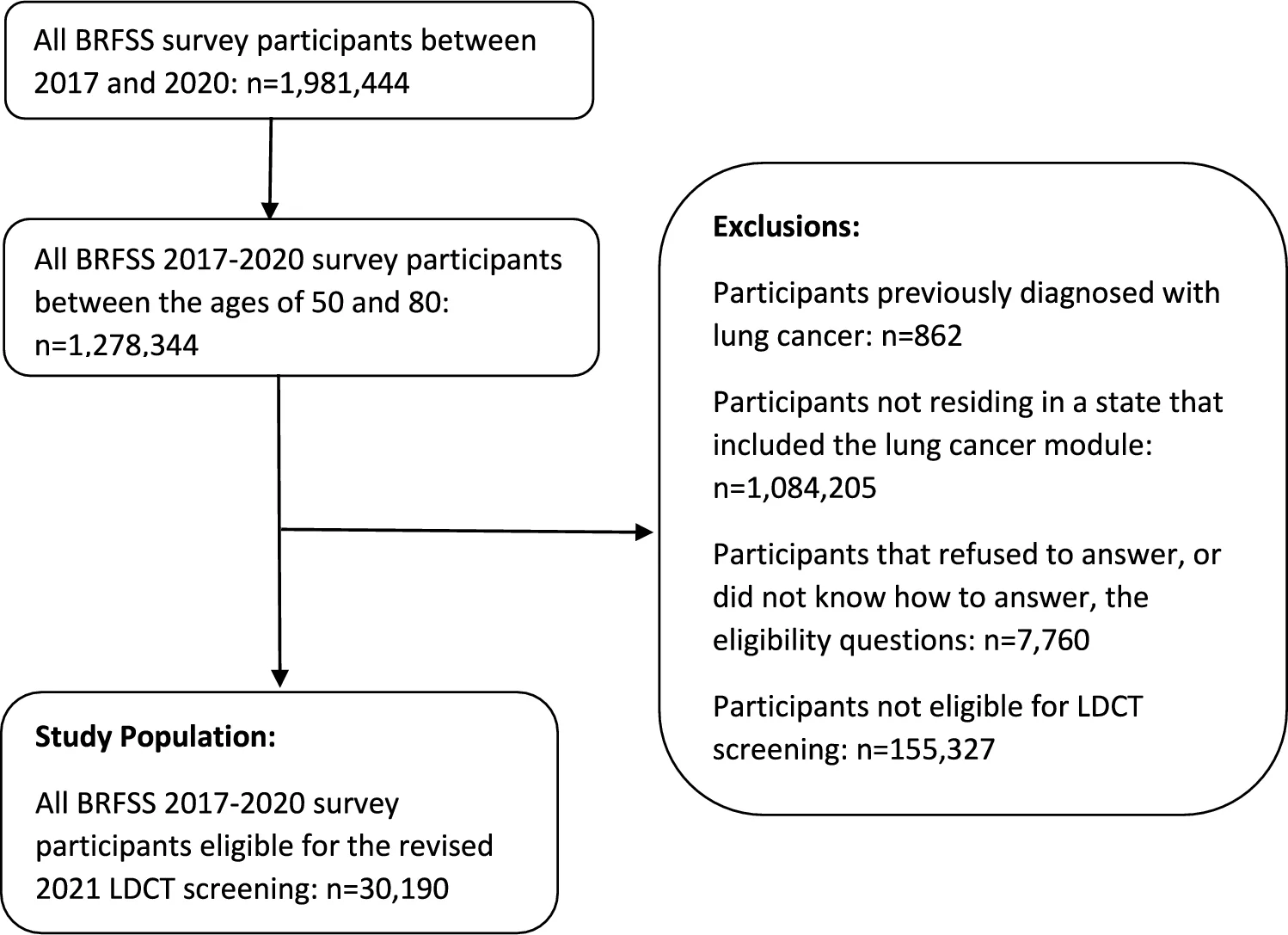
Study population flow diagram

Figure 2 summarizes the application of the inclusion and exclusion criteria used to define our study population. From 2017 to 2020, a total of 30,190 survey participants from up to 23 different states met the eligibility criteria annually: 8,720 from 12 states in 2017, 5,494 from 9 states in 2018, 13,074 from 23 states in 2019, and 2,902 from 5 states in 2020. From this study population, we determined eligibility for LDCT screening according to the 2013 USPSTF recommendations and newly eligible for LDCT screening under the 2021 USPSTF recommendations using age, self-reported smoking, and smoking cessation history (see Sect. "Measurements").

### Statistical analysis

Data management and analysis were conducted in SAS Version 9.4 (SAS Institute; Cary, NC). All analyses used the raking-derived weighting variable provided by BRFSS to account for the survey’s complex sampling design. Proc surveyfreq was used to analyze the data using the BRFSS strata variable (_ststr) and BRFSS cluster variable (_psu). Since data were used across multiple years of BRFSS, the weighting variable was adjusted proportionally using the sample size from each year. For each study variable, the proportion of participants within each strata were calculated separately for those eligible under the 2013 recommendations and those newly eligible under the 2021 recommendations. Chi-squared tests were calculated to determine if there was a statistically significant difference (*p* < 0.05) in key variables between those eligible based on the 2013 recommendations and those who became newly eligible based on the changes in the 2021 recommendations. We also calculated the percent increase in eligibility by dividing the weighted frequency of the newly eligible under the 2021 recommendations by the originally eligible under the 2013 recommendations. This study did not require Institutional Review Board oversight as only public use data were used, and therefore the IRB determined that the study did not meet the criteria for human subjects research.

## Results

### Participant demographics

Demographic data for the study population is shown in Table 1. All percentages are weighted, but all frequencies are unweighted and represent the actual number of survey participants. There were 30,190 survey participants eligible under the 2013 and/or 2021 USPSTF recommendations. There were 19,898 participants (61.6%) who were eligible for screening under the 2013 recommendations and 10,292 participants (38.4%) who became newly eligible under the 2021 recommendations. Thus, based on absolute frequencies an estimated 62.4% of participants were newly eligible due to the new 2021 recommendations.

**Table 1:** Comparison of sociodemographic characteristics of persons eligible for LDCT screening for lung cancer according to 2013 recommendations vs. 2021 recommendations only, BRFSS data from 23 states, 2017–2020

	Total population	Eligible for LDCT screening: 2021 recommendations (i.e., the Total)		Eligible for LDCT screening: 2013 recommendations		Newly eligible for LDCT screening: 2021 recommendations		Weighted Frequency % Increase ^a,b^	P-value^c^
	N	%^a^	95% CI	%^a^	95% CI	%^a^	95% CI		
All	30,190	100%	N/A	61.60%	(60.4%, 62.8%)	38.40%	(37.2%, 39.6%)	62.40%	
Age group (years)^d^									
55 to 59	5,480	24.70%	(23.6%,21.2%)	24.10%	(22.9%,25.4%)	26.30%	(24.0%,28.5%)	36.40%	< 0.0001
60 to 64	6,630	28.80%	(27.6%,30.0%)	28.50%	(27.2%,29.9%)	29.50%	(27.0%,31.9%)	34.60%	
65 to 69	5,840	19.10%	(18.1%,20.1%)	19.10%	(17.9%,20.2%)	19.30%	(17.2%,21.3%)	33.90%	
70 to 74	4,479	14.80%	(13.9%,15.7%)	14.60%	(13.5%,15.6%)	15.50%	(13.5%,17.5%)	35.60%	
75 to 80	4,145	12.60%	(11.8%,13.5%)	13.70%	(12.7%,14.7%)	9.50%	(8.2%,10.9%)	23.30%	
Sex									
Male	14,933	53.60%	(52.4%,54.8%)	55.90%	(54.4%,57.4%)	49.90%	(47.8%,51.9%)	55.60%	< 0.0001
Female	15,241	46.40%	(45.2%,47.6%)	44.10%	(42.6%,45.6%)	50.10%	(48.1%,52.2%)	70.80%	
Race and ethnicity									
Non-Hispanic White	26,127	84.50%	(83.5%, 85.5%)	86.40%	(85.2%, 87.6%)	81.50%	(79.6%, 83.4%)	58.80%	0.0005
Non-Hispanic Black	1,393	7.60%	(6.8%, 8.4%)	6.60%	(5.7%, 7.5%)	9.20%	(7.8%, 10.5%)	86.30%	
Hispanic	530	3.40%	(2.7%, 4.0%)	2.70%	(2.0%, 3.5%)	4.40%	(3.1%, 5.7%)	100%	
Other (Indigenous, Asian, multi-race, etc.)	1,660	4.50%	(4.0%, 5.0%)	4.30%	(3.7%, 4.8%)	5.00%	(4.1%, 5.8%)	72.60%	
Marital status									
Divorced/Separated	8,651	26.70%	(25.6%,27.8%)	27.10%	(25.7%,28.4%)	26.10%	(24.3%,27.8%)	60.00%	< 0.0001
Married	12,790	47.80%	(46.6%,49.0%)	47.50%	(46.0%,49.0%)	48.30%	(46.2%,50.4%)	63.40%	
Never married	2,449	8.10%	(7.3%,8.8%)	6.60%	(5.9%,7.4%)	10.40%	(9.0%,11.8%)	97.40%	
Unmarried couple	747	2.90%	(2.4%,3.3%)	2.50%	(2.0%,3.0%)	3.40%	(2.7%,4.1%)	84.40%	
Widowed	5,403	14.60%	(13.8%,15.4%)	16.30%	(15.2%,17.4%)	11.80%	(10.6%,13.1%)	45.20%	
Sexual orientation									
Straight	15,689	95.70%	(95.1%, 96.3%)	95.90%	(95.2%, 96.6%)	95.30%	(94.2%, 96.3%)	62.00%	0.76
Lesbian/Gay	312	1.90%	(1.4%, 2.3%)	1.70%	(1.2%, 2.3%)	2.10%	(1.3%, 2.8%)	75.10%	
Bisexual	203	1.10%	(0.9%, 1.4%)	1.10%	(0.8%, 1.4%)	1.20%	(0.7%, 1.7%)	71.80%	
Something else/Not Sure	257	1.30%	(1.0%, 1.6%)	1.20%	(0.9%, 1.6%)	1.40%	(0.9%, 1.9%)	70.90%	
Educational status									
Less than high school	3,328	19.20%	(18.1%,20.3%)	18.80%	(17.5%,20.1%)	19.80%	(17.8%,21.8%)	65.90%	0.1998
High school graduate	11,505	37.10%	(36.0%, 38.3%)	37.40%	(36.0%, 38.8%)	36.70%	(34.7%, 38.7%)	61.40%	
Some college/Associate’s degree	9,541	31.70%	(30.6%, 32.8%)	32.30%	(30.9%, 33.7%)	30.80%	(28.9%, 32.7%)	59.70%	
Bachelor’s degree or higher	5,757	12.00%	(11.4%, 12.6%)	11.50%	(11.6%, 13.8%)	12.70%	(11.6%, 13.8%)	68.70%	
Employment									
Not retired	17,266	63.90%	(62.8%, 65.0%)	56.10%	(54.6%, 57.5%)	76.50%	(74.8%, 78.1%)	85.10%	< 0.0001
Retired	12,813	36.10%	(35.0%, 37.2%)	43.90%	(42.5%, 45.4%)	23.50%	(21.9%, 25.2%)	33.40%	

^a^Percentages are weighted using survey weights

^b^Calculated by dividing the weighted frequency of the newly eligible under 2021 recommendations by the originally eligible under the 2013 recommendations

^c^Bolded items indicate statistical significance at alpha = 0.05 level

^d^All values in age group calculated using only individuals between 55 and 80

### Differences in population eligible for lung cancer screening under the 2013 and newly eligible under 2021 recommendations

Comparisons of the populations eligible for lung cancer screening under the 2013 recommendations and the new 2021 recommendations revealed statistically significant differences, including the distribution of eligible participants by age group, sex, race and ethnicity, marital status, and retirement status (all *p*-values < 0.001) (Table 1). By age group (*p* < 0.01), the percentage of eligible participants between the ages of 75 and 80 was lower among the newly eligible than the originally eligible (9.5% vs 13.7%). By sex, the percentage of eligible females was greater among the newly eligible than the originally eligible (50.1% vs 44.1%), resulting in a 70.8% numerical increase in eligible females due to the new 2021 recommendations (*p* < 0.01). By race and ethnicity (*p* < 0.01), the percentage of eligible non-Hispanic White participants was lower among the newly eligible than the originally eligible (81.5% vs 86.4%), the percentage of non-Hispanic Black participants was greater among the newly eligible than the originally eligible (9.2% vs 6.6%), and the distribution of Hispanic participants was greater among the newly eligible than the originally eligible (4.4% vs 2.7%). The number of eligible participants increased by 86.3% for non-Hispanic Black participants and 100% for Hispanic participants due to the new 2021 recommendations (*p* < 0.01). By marital status (*p* < 0.01), the percentage of eligible never married participants was greater among the newly eligible than the originally eligible (10.4% vs 6.6%), but the percentage of eligible widowed participants was lower among the newly eligible than the originally eligible (11.8% vs 16.3%). The number of eligible participants increased by 97.4% for never married participants and 84.4% for unmarried participants due to the new 2021 recommendations (*p* < 0.01). By retirement status (*p* < 0.01), the percentage of eligible non-retired participants was greater among the newly eligible than the originally eligible (76.5% vs 56.1%), resulting in an 85.1% numerical increase in non-retired participants due to the new 2021 recommendations (*p* < 0.01). No statistically significant differences were noted for sexual orientation (*p* = 0.76) or education level (*p* = 0.20).

### Health-related variables

Statistically significant differences were observed for respiratory comorbidities, vaccination status, and presence of cardiovascular comorbidities between those eligible under the 2013 recommendations and those newly eligible under the 2021 recommendations (Table 2). By respiratory status (*p* < 0.01), the percentage of participants with no respiratory comorbidities was greater among the newly eligible than the originally eligible (67.8% vs 60.7%) and the percentage of participants with COPD/emphysema/chronic bronchitis and not asthma was greater among the newly eligible than the originally eligible (24.2% vs 16.3%), resulting in a 88.4% numerical increase in persons with asthma without COPD/emphysema/chronic bronchitis due to the new 2021 recommendations (*p* < 0.01). There were 394 individuals who did not know their respiratory comorbidity status and 18,395 who responded that they had neither. By vaccination status (*p* < 0.01), the percentage of non-vaccinated participants was greater among the newly eligible than the originally eligible (43.3% vs 35.7%), resulting in a 79.9% numerical increase in non-vaccinated participants due to the new 2021 recommendations (*p* < 0.01). By cardiovascular comorbidity status (*p* < 0.01), the percentage of participants with at least one cardiovascular comorbidity was lower among the newly eligible than the originally eligible (20.5% vs 27.1%).

**Table 2:** Comparison of health-related variables of persons eligible for LDCT screening for lung cancer according to 2013 recommendations vs. 2021 recommendation only, BRFSS data from 223 states, 2017–2020

	Total population	Eligible for LDCT screening: 2021 recommendations (i.e., the Total)		Eligible for LDCT screening: 2013 recommendations		Newly eligible for LDCT screening: 2021 recommendations		Weighted Frequency % Increase ^a,b^	P-value^c^
	N	%^a^	95% CI	%^a^	95% CI	%^a^	95% CI		
Respiratory comorbidities									
Neither/Not Sure/Do Not Know	18,789	63.40%	(62.2%, 64.6%)	60.70%	(59.2%, 62.1%)	67.80%	(65.8%, 69.7%)	69.70%	< 0.0001
Both	3,103	10.30%	(9.5%, 11.0%)	10.60%	(9.8%, 11.5%)	9.60%	(8.3%, 10.9%)	56.40%	
Asthma	1,484	5.20%	(4.7%, 5.7%)	4.50%	(3.9%, 5.1%)	6.30%	(5.4%, 7.3%)	88.40%	
COPD/Emphysema/Chronic bronchitis	6,814	21.20%	(20.2%, 22.2%)	24.20%	(23.0%, 25.5%)	16.30%	(14.7%, 17.8%)	41.90%	
Vaccination status									
Pneumonia vaccination	5,565	17.40%	(16.4%, 18.3%)	18.00%	(16.8%, 19.2%)	16.40%	(14.9%, 17.9%)	56.80%	< 0.0001
Flu vaccination	3,678	13.10%	(12.2%, 13.9%)	12.50%	(11.5%, 13.5%)	14.00%	(12.6%, 15.5%)	70.20%	
Both	10,689	32.10%	(31.0%, 33.2%)	35.70%	(34.4%, 37.1%)	26.30%	(24.4%, 28.2%)	45.90%	
Neither/Not Sure/DNK/Refused	10,258	37.50%	(36.3%, 38.7%)	33.80%	(32.4%, 35.3%)	43.30%	(41.2%, 45.4%)	79.90%	
Presence of cardiovascular comorbidities									
Yes	7,658	24.60%	(23.5%, 25.6%)	27.10%	(25.8%, 28.4%)	20.50%	(18.9%, 22.2%)	47.30%	< 0.0001
No	22,532	75.40%	(74.4%, 76.5%)	72.90%	(71.6%, 74.2%)	79.50%	(77.8%, 81.1%)	67.90%	

^a^Percentages are weighted using survey weights

^b^Calculated by dividing the weighted frequency of the newly eligible under 2021 recommendations by the originally eligible under the 2013 recommendations

^c^Bolded items indicate statistical significance at alpha = 0.05 level

### Geographic variables

State differences between those eligible under the 2013 recommendations and those newly eligible under the 2021 recommendations were not statistically significant (*p* = 0.28). Results regarding the region and urban/rural status are shown in Table 3. The difference in urban/rural status was statistically significant (*p* < 0.01), with a larger proportion of urban residents being covered under the new 2021 recommendations (88.3% vs 85.9%). No statistically significant differences were noted for U.S. census region (*p* = 0.93).

**Table 3:** Comparison of geographic variables of persons eligible for LDCT screening for lung cancer according to 2013 recommendations vs. only 2021 recommendations

	Total population	Eligible for LDCT screening: 2021 recommendations (i.e., the Total)		Eligible for LDCT screening: 2013 recommendations		Newly eligible for LDCT screening: 2021 recommendations		Weighted Frequency % Increase ^a,b^	*p*-value^c^
	N	%^a^	95% CI	%^a^	95% CI	%^a^	95% CI		
Region									
Midwest	7,388	20.00%	(19.6%, 20.4%)	20.10%	(19.4%, 20.8%)	19.90%	(18.8%, 21.1%)	61.70%	0.9299
Northeast	6,930	18.10%	(17.5%, 18.6%)	18.00%	(16.9%, 19.0%)	18.20%	(16.8%, 19.5%)	63.10%	
South	12,588	53.00%	(52.3%, 53.7%)	53.10%	(52.0%, 54.3%)	52.80%	(51.0%, 54.6%)	61.90%	
West	3,284	8.90%	(8.6%, 9.2%)	8.80%	(8.3%, 9.3%)	9.10%	(8.3%, 9.9%)	64.90%	
Urban/Rural^d^									
Urban	15,646	86.80%	(86.1%, 87.6%)	85.90%	(84.9%, 86.9%)	88.30%	(87.7%, 90.2%)	65.90%	0.0038
Rural	5,824	13.20%	(12.4%, 13.9%)	14.10%	(13.1%, 15.1%)	11.70%	(9.8%, 12.3%)	53.30%	

^a^Percentages are weighted using survey weights

^b^Calculated by dividing the weighted frequency of the newly eligible under 2021 recommendations by the originally eligible under the 2013 recommendations

^c^Bolded items indicate statistical significance at alpha = 0.05 level

^d^Data available only for 2018–2020

## Discussion

Using BRFSS data from 23 states over several years provided a large dataset to quantify the impact of the USPSTF 2021 change in recommended eligibility criteria for LDCT screening for lung cancer compared to the prior 2013 USPSTF recommendations. Importantly, the results of the present study corroborated prior studies of this topic [15, 16, 33], indicating that the revision of LDCT screening recommendations in 2021 resulted in an approximately 60% increase in the absolute number of people eligible for LDCT screening.

The participants more likely to be newly eligible under the 2021 recommendations included those who identify as female, Black, and Hispanic and those who have never been married and/or are not retired. The participants more likely to be newly eligible under the 2021 recommendations also included those who have not been diagnosed with asthma or COPD/emphysema/chronic bronchitis, have been diagnosed with asthma but not COPD/emphysema/chronic bronchitis, received the flu vaccine (or do not get any vaccines), and/or have no cardiovascular comorbidities. Urban-dwelling people also had significantly increased eligibility for screening under the new 2021 recommendations. Thus, our results replicate the findings that the changes in recommendations were successful in reducing the disparities in eligibility by sex, race, and COPD diagnosis as shown in previous studies [10–16], and they provide new insights into the downstream impact of the change in recommendations on characteristics other than sex, race, and COPD diagnosis that were not previously considered.

A previously mentioned study found a relative increase of 13.8% for women as opposed to men in the newly eligible population, and our study confirmed this result by finding a relative increase of 15.2% for women as opposed to men [16]. The increase in eligibility for women is important because more women than men are expected to develop lung cancer in 2023 [1]. The same study found a relative increase of 20.7% for non-Hispanic Black persons compared with non-Hispanic White persons; we observed an even greater increase of 27.5% [16]. The increase in eligibility of Black persons is of public health importance because they have higher incidence and mortality of lung cancer than any other racial or ethnic group, and they were previously less likely to be eligible since they have a lower exposure to smoking than other groups [34]. Expanded access to lung cancer screening for Black individuals holds promise for reducing this longstanding racial disparity in lung cancer mortality.

Eligibility disparities among other groups also decreased due to the revised recommendations. The decrease in eligibility disparities by marital status is an important change since individuals who have never been married have a 12% higher lung cancer mortality than those who have been married [35] and only an 8.4% five-year survival rate [18]. Married and widowed cancer patients are also more likely than never married cancer patients to have treatable types of cancer [18]. The recommendation changes greatly increase the eligibility of never married individuals. These benefits from expanded eligibility did not extend to divorced/separated individuals in our study, however.

Asthma is a clinical risk indicator for developing lung cancer [36] and for poorer lung cancer survival [37]. It is thus reassuring people with self-reported asthma experienced decreased eligibility disparities due to the LDCT recommendation change. We are hesitant to make strong inferences based on self-reported respiratory conditions, but to the extent the observed increased eligibility of people living with asthma reflects a genuine change due to the revised recommendations, this is a positive development. Improving patient-provider communication and community engagement around preventive services, broadly defined, may improve both LDCT screening AND vaccination uptake as previously seen in other studies attempting to “bundle” preventive health services (e.g., FluFIT) [38].

Some of these changes between the groups eligible for LDCT screening were expected. Since the minimum age requirement changed from 55 to 50, it was not surprising to see that eligibility among non-retired participants increased by 85.1%. Also, the minimum age change increased eligibility for younger and healthier individuals, as 46.3% of those eligible under the new recommendations were between the ages of 50 and 55, and the eligibility of those with no respiratory or cardiovascular comorbidities increased by 69.7% and 67.9%, respectively. This is an important group to have an increased eligibility since the benefit of early lung cancer detection and treatment is greatest among young individuals. However, some changes in eligibility we expected to see did not occur, such as with regards to the participants’ census region. We expected to see increased eligibility in the West as compared to other regions since smoking frequency is lower in the West; the West and Northeast regions did have the greatest increases in eligibility, but the increases were only slightly greater than the South and Midwest regions and the overall comparison between regions was not statistically significant [39].

Unfortunately, some eligibility disparities remain, as the 2021 recommendation changes did not increase eligibility in all at-risk groups. This is an important finding because eligibility needs to be higher among groups who have a higher risk of lung cancer. The recommendation changes did not significantly increase eligibility among non-straight participants. This is not surprising since non-straight participants tend to smoke more than straight participants and the recommendation changes were meant to target individuals who smoke less [20]. Future discussion should focus on increasing eligibility for non-straight participants since they have a higher risk of lung cancer [20]. Another group that still experiences eligibility disparities is individuals with lower education levels [40]. It was suggested that they were less likely to be eligible due to the age range that was previously recommended by the USPSTF [40]. However, we found that even with the minimum age change from 55 to 50 years old, eligibility did not increase much among persons whose highest educational attainment was a high school diploma or less compared to participants with higher levels of educational attainment. Future discussion should be aimed at increasing eligibility among individuals with lower education levels and individuals who are 65 and older since that age group has been reported to have lower levels of high school and college attainment than those younger than 65 [41]. Doing so would also help target those most likely to be diagnosed with lung cancer since lung cancer is most likely to occur in individuals who are 65 and older, with 70 being the average age of being diagnosed with lung cancer [1].

Strengths of this study included it being one of the first studies to investigate the differences between those eligible only under the old 2013 recommendations and those newly eligible under the new 2021 recommendations other than just sex, race and ethnicity, income, and COPD diagnosis. The dataset analyzed had a large sample size. The study’s external validity is enhanced with the inclusion of data from 23 states, but is not as strong as it would have been if data from more states could have been included. The results reinforced the value of this line of inquiry, as previously unstudied factors were found to be impacted by the 2021 recommendation changes.

Relying on existing state-level data from the BRFSS led to the strengths noted above, but this approach also introduced limitations due to missing data from some states and for certain variables as described below. The net result of these limitations was a diminished likelihood that the results are generalizable to the US as a whole and a reduced sample size, and thus statistical precision, for certain variables. Not all states were included because the BRFSS lung cancer module was optional and so 27 states were not included in this study. Thus, while the inclusion of 46% of US states is a strength, the possibility remains that if data were available from all 50 states the results could differ from the observed results. Furthermore, since the response rate ranges from 31 to 64%, nonresponse could also lead to the true results being different from the observed results. With respect to specific variables, sexual orientation, race, and prior lung cancer history deserve mention. The sexual orientation question was optional, and thus only 16,461 of the 30,190 total participants were included in the analysis of sexual orientation. The participants in our analysis were predominantly White Non-Hispanic, which makes changes in the proportion of screening eligibles by race and ethnicity difficult to interpret. Furthermore, only 1.6% of the US population consists of indigenous persons [42], and therefore the sample size in the BRFSS was insufficient to place them in a race category of their own. As a result, we placed them in the “Other” category with Asians and multi-race persons, which unfortunately obscures the varying eligibility changes between them. Items regarding personal cancer history were part of an optional module separate from the lung cancer module so that we could only identify and exclude lung cancer patients from the 9 states that included the cancer survivorship module and the lung cancer screening module in the same year. Furthermore, this module only asks participants about their first cancer diagnosis so if a participant was diagnosed with lung cancer after an initial diagnosis of another cancer type, they may have been included in the study despite the previous lung cancer diagnosis. Despite the theoretical possibility of participants with a personal history of lung cancer being included in the study population, given the low prevalence of lung cancer in the general population, the number of actual participants with a lung cancer diagnosis would be expected to be negligible. Furthermore, the urban/rural status data were only available between 2018 and 2020, thus information from 2017 was lacking. Other questions were also missing from some years, such as high blood pressure and high cholesterol status, which limited the types of variables we could include. Finally, for BRFSS 2019 and 2020, there is no question asking the participants how many years total they have smoked, so we estimated this by subtracting their age, or the age they were when they last smoked if they smoked previously, by the age they were when they first started smoking. This approach has the potential to overestimate the duration of smoking because participants could have quit smoking and then started smoking again later. In BRFSS 2017 and 2018, an item asked how many years total the participant had smoked. To estimate the validity and reliability of this estimate, we calculated the pack-year history for the 2017 and 2018 participants in two different ways, once by using the question that asked how many years total they smoked and once by calculating the total years smoked by subtracting the age they were when they first started smoking by the age they were when they last smoked. We calculated the difference between the pack-year histories using the two different methods and estimated an average difference of 5.6 years. Furthermore, the differences between the two methods led to conflicting results regarding the eligibility of 14.8% of the participants in 2017 and 2018 (Supplementary Tables).

## Conclusions

In conclusion, the results of this study indicate the revision of the LDCT screening recommendations decreased eligibility disparities in sex, race, and ethnicity. The revisions also expanded eligibility based on marital status, respiratory comorbidities, and vaccination status which could lead to increased survival rates among these groups. Eligibility disparities were not impacted for educational level.

Expanding the eligibility criteria is an important step, but it will not have a population impact if those newly eligible do not get screened. Thus, a key question for future research is to determine whether those who are newly eligible for screening are more likely to undergo LDCT screening. Henderson and colleagues [15] addressed this question and the results are concerning, indicating that those newly eligible for screening are substantially less likely to be screened than those who remained eligible. For example, individuals who were never married have previously been observed to be less likely to receive LDCT screening than other marital status groups. Even though we observed that the recommendation changes increased eligibility for this group, it does not mean that utilization will necessarily increase for this group [43]. This is also true for younger individuals without physical health conditions, such as respiratory and cardiovascular comorbidities, since it has been found that they are less likely to participate in cancer screenings [44]. Therefore, futures studies should investigate how to increase utilization among groups with higher lung cancer mortality, such as never married individuals, younger individuals, and individuals without physical health conditions.

## Supplementary Information

Below is the link to the electronic supplementary material.

Supplementary file1 (DOCX 14 kb)

## Data Availability

All datasets used for analysis are available via the BRFSS website: https://www.cdc.gov/brfss/
