# Correction: Comparison of the characteristics of the population eligible for lung cancer screening under 2013 and population newly eligible under 2021 US Preventive Services Task Force recommendations

**DOI:** 10.1007/s10552-026-02245-x

**Published:** 2026-09-26

**Authors:** Nicholas Yell, Jan M. Eberth, Anthony J. Alberg, Peiyin Hung, Mario Schootman, Alexander C. McLain, Reginald F. Munden

**Affiliations:** 1 Department of Health Services Policy and Management, University of South Carolina Arnold School of Public Health, Columbia, SC USA; 2 Department of Health Management and Policy, Drexel University Dornsife School of Public Health, Philadelphia, PA USA; 3 Department of Epidemiology and Biostatistics, University of South Carolina Arnold School of Public Health, Columbia, SC USA; 4 Department of Internal Medicine, University of Arkansas for Medical Sciences, Little Rock, AR USA; 5 Department of Radiology and Radiological Sciences, Medical University of South Carolina, Charleston, SC USA

**Correction to: Cancer Causes & Control (2024) 35:1233-1243**

**https://doi.org/10.1007/s10552-024-01880-6**

In this article, the column header ‘Change’ in Tables 1 and 2 and ‘Weighted % Increase’ in Table 3 were incorrect and should have read ‘Weighted Frequency % Increase a,b’.

Footnote b ‘Gives the increased percentage of eligible individuals within each category level’ of all tables was incorrect and should have read ‘Calculated by dividing the weighted frequency of the newly eligible under 2021 recommendations by the originally eligible under the 2013 recommendations’. In the sentence in ‘method section’ in this article, ‘We also calculated the percent increase in eligibility due to the new 2021 recommendations’ should have read ‘We also calculated the percent increase in eligibility by dividing the weighted frequency of the newly eligible under the 2021 recommendations by the originally eligible under the 2013 recommendations’.

The original article has been corrected.

