# Supplementary material for: Comparison of the characteristics of the population eligible for lung cancer screening under 2013 and population newly eligible under 2021 US Preventive Services Task Force recommendations
Source: Cancer Causes Control. 2024 May 8;35(9):1233–43. doi: 10.1007/s10552-024-01880-6 (PMC11377476; doi:10.1007/s10552-024-01880-6)
Supplement: Supplementary file 1 — Supplementary file1 (DOCX 14 kb) [file 10552_2024_1880_MOESM1_ESM.docx]

**Supplementary Data: Eligibility of Participants from 2017 and 2018 Compared Using the Two Different Smoking Length Methods**

**Panel A**

| **2013 Recommendations** | **Total** | **Eligible Using Age Variables** | **Not Eligible Using Age Variables** | **Total** |
| --- | --- | --- | --- | --- |
| Eligible Using Years Smoked Variable | 2,449 | 31.4% | 2.1% | 33.5% |
| Not Eligible Using Years Smoked Variable | 4,868 | 6.0% | 60.5% | 66.5% |
| Total | 7,317 | 37.4% | 62.6% | 100% |

**PanelB**

| **2021 Recommendations** | **Total** | **Eligible Using Age Variables** | **Not Eligible Using Age Variables** | **Total** |
| --- | --- | --- | --- | --- |
| Eligible Using Years Smoked Variable | 3,578 | 46.4% | 2.5% | 48.9% |
| Not Eligible Using Years Smoked Variable | 3,739 | 6.8% | 44.3% | 51.1% |
| Total | 7,317 | 53.1% | 46.9% | 100% |
